# The Fatty Heart

**Published:** 1889-01

**Authors:** E. H. Kisch

**Affiliations:** Marienbad


### THE FATTY HEART.

Professor E. H. Kisch, Marienbad.

Macroscopically we see the fat penetrate, in the form of yellow streaks, the intermuscular tissue of the myocardium, which turns into a yellowish-brown color, and becomes soft and friable.

Microscopical examination reveals the cells of the subpericardial fatty tissue appear larger than normal, containing more arterial and venous blood-vessels than in the normal state. One sees the fat-cells around the blood-vessels and in the pericardium accompanying them in their course and penetrating the muscular substance; the muscular fibres are pushed asunder, they proliferate between them, and enlarge the interstices between the muscular fibrillæ, which thus become compressed. It can be seen best on the right ventricle, where, between the muscular fibres, larger or smaller diffused fatty foci are situated. The muscular fibres are commonly well preserved, with normal horizontal striation, and only degenerated where the interstices are greatly enlarged by fat. With stronger lenses, the lines are then only observed in very fine granules, strongly shining. In some muscular fibres the horizontal striation is lost, one sees only longitudinal fibres consisting of strongly shining fatty drops.

We have, therefore, as the anatomical result of a fatty heart: *Fat penetrating the subpericardial connective tissue, lesion of the structure of the heart during its further course, decrease of the contractile substance and molecular changes of the muscular fibres.*

The function of a fatty heart becomes mechanically obstructed, because the layer of fat proliferating from the subpericardial connective tissue and nearly surrounding the heart prevents the motions of the heart. Another functional disturbance is also caused by the penetration of the fat into the interstitial connective tissue of the myocardium; thus the mechanical formation of the heart is lessened and its power diminished. A further increase of fat causes a partial atrophy of the muscular walls of the heart by pressure. In consequence thereof, the structural firmness of the heart is shaken, and its result is dilatation of one or more cavities or of the whole heart, and with increased labor the heart may become insufficient. In all cases of obesity, much more is required from the heart than in the normal state. The increase of the fatty tissue in the panniculus adiposus, mesenterium, omentum, etc., causes new blood-vessels to spring up, which, corresponding to the increased weight of the body through the formation of fat, necessitates increased labor for the circulation of the blood.

Where the increase of fat in the body is only a gradual one, and where its accumulation does not reach extreme degrees, the fatty heart, only moderately surrounded by fat, may still be able to perform its increased labors for some time. The *first stage* of the fatty heart shows only slight troubles. Only when the patient over-exerts himself bodily by running, ascending, stooping, and similar locomotions, or after a copious meal, some dyspnœa and slight palpitation may set in. He still sleeps good and has no asthma. Objectively the examination of the heart shows nothing abnormal, only the sounds of the heart sometimes appear rather dull, and the dullness may extend over a larger space than normal, and the beat of the apex is found lower. An intensive dullness over the sternum shows a copious accumulation of fat in the mediastinal cellular tissue. In its further course, and by a more copious increase of fat throughout the body, the increased activity of the fatty heart, in connection with increased resistance from the extension of the abdomen, pushing upward of the diaphragm and diminution of the thoracic space leads to thickening and dilatation of the left ventricle, and when this hypertrophy and dilatation do not suffice for compensation, it must lead, in consequence of the over-filling of the venous system, finally to dilatation and hypertrophy of the right ventricle. During the *second stage* of the fatty heart, we meet palpitation and increased dyspnœa in ascending or longer bodily exercise. Severe dyspnœa and vertigo appear; at night asthmatic attacks. The fat patient wakes up several times during the night, must rise from his bed to breathe. Objectively we find the dullness of the heart increased in length as well as in breadth, the impulse of the heart is mostly diffuse, and the apex felt more outwardly. Auscultation reveals pure, but dull, sounds, sometimes they are loud and clear, or we may hear with the systole a short blowing or a double sound. The pulse may be frequent or retarded, or full, large, tense. The more difficult circulation of the blood, in consequence of the diminished propelling power of the heart, shows itself all through the venous system, as by dilatation of the superficial cutaneous veins, by varicose in the lower extremities, and especially by hemorrhoids. When such a lipomatosis universalis is not kept within bounds by rational treatment, the *third stage* sets in, fatty degeneration of the myocardium takes place, with all the symptoms of insufficiency. Dyspnœa is now nearly constant, with palpitations and præcordial anguish. The dullness of the percussion sound is heard over a wide extent, the impulse of the heart weak, the sounds of the heart dull, the second sound of the aortic valve more strongly marked. The weakness of the heart shows itself by stagnation of blood in the lungs, and from there backwards in the large veins of the body, and cyanosis follows. We meet now cardial asthma, torturing cough, seething in chest with foaming, bloody mucus. Paroxysmally angina pectoris, with other neuralgic pains, often a pain radiating from the cardiac region toward the left shoulder. Finally, this stagnation in the larger circulation leads to cerebral symptoms, to serous effusions in the subcutaneous cellular tissue, to affections of the liver, spleen, and kidneys. Albumen in the urine and œdema pedum are usual manifestations, and a fatal issue might be expected, often with the symptoms of œdema pulmonum or a pneumonia by stasis. Paralysis of the heart may also suddenly set in, and the patient succumbs.

REMARKS.—Professor Kisch, in his Marienbad, has many opportunities to treat obesity, and thus, also, the fatty heart, and with his patients the *cur*, as the German call the strictly prescribed diet and the regulation of the drinking of the mineral waters of this celebrated spring, will certainly benefit them during their sojourn; but how few keep up this regular mode of living after returning home, and thus necessitate a yearly pilgrimage to that shrine, which reduces the amount of fat which they are obliged to carry about. The same may be said of Professor Oertel's treatment at Meran, Tyrol, who relies on active exercise, regulated by the physicians, and ascending hills and mountains in this Alpine region, so rich in oxygen, and thus quickening respiration and consuming heat-producing tissue.

Arndt has a very good article on this fatty heart in the first volume of his *Encyclopædia*, and as Phosphorus, Arsenious acid, Sulphuric acid, Plumbum, Antimony, and Alcohol have the power to produce *fatty degeneration of the heart*, he recommends them in the treatment of this disease; but the *fatty heart* in its first and second stage will hardly be benefited by these powerful drugs, and its third stage is nigh incurable. He mentions Calcarea, Graphites, Lycopodium, and Ferrum phosphoricum, in minute doses, to antidote the tendency to adiposus, and we agree fully with him that more can be gained by constitutional treatment than by drugs whose abuse lead to degenerative processes.

Leucophlegmacy, malassimilation, and obesity are all found in the pathogenesis of the salts of Lime, and especially under *Calcarea ostrearum.* Hahnemann, in his *Chronic Diseases*, II, 255, makes here a valuable remark: "In affections of persons advanced in age, Calcarea, even after other intermediate remedies, can scarcely be repeated with advantage; a dose which is given after another, without any previous intermediate remedy, is almost always prejudicial. In cases of children, however, several doses may be given in succession, provided the remedy continues to be indicated, the younger the children the more frequently the remedy may be repeated. And when we look with its dyspeptic and flatulent symptoms to such indications as interruption of breath when walking in the wind, then in the rooms dyspnœa, which increases as soon as he walks a few steps; frequent necessity of deep breathing; shortness of breath, worse when sitting and in motion; the breath becomes short upon ascending the least height; the whole chest is intensely painful when touched or during an inspiration; pain in front of the chest, as if it were pressed upon; oppressive anguish in the chest; palpitation of the heart, with excessive anguish, etc., we get here a full picture of the first stages of the fatty heart, and its judicious use will prevent it reaching its third stage." But remember Hahnemann's advice of Calcarea when prescribed to people old in years, for there is also a precocious old age, and many a one who ought to be in the zenith of life has used up his life-force prematurely, and I doubt whether for such senility Calcarea will be the remedy.

Here the *salts of Barium* come in, which is considered a valuable remedy for senility, so far as this is *premature, and therefore morbid.* Hughes's *Pharmaco-dynamics,* fourth ed., and Noack and Trinks teach that Baryta is especially suitable for the affections of first childhood, and more particularly still for those of old age, when there is mental and physical weakness—*a marasmus senilis.* We meet here many symptoms similar to Calcarea, as fullness of chest, with short breath, especially when ascending a height, and with stitches in the chest when inspiring; the pain in the chest is partly relieved by eructations; pressive heaviness across the chest, increased by inspiration and then causing painful stitches under the upper extremity of the sternum; throbbing in the left side of the chest, commencing at the pit of the stomach and ascending; palpitation when lying on left side.

Whereas we meet in Arsenicum and Phosphorus fatty degeneration and destruction of the muscular fibres of the heart, we prefer *Aurum muriaticum* for the atheromatous condition of the arteries (coronary) so often found in ripe old age, with an obesity of the heart, the fat being imbedded in the connective tissue around and through the heart between its muscular fibres, but without destroying its structure; frequent attacks of anguish about the heart, with tremulous fearfulness and faintings; violent heart-stroke, not synchronous with the pulse; constantly compelled to be in motion, but, when riding or walking, palpitations compel him to stop, sometimes with irregular, intermittent pulse and dyspnœa.

Under *Bromide of Potassium*, we detect some symptoms which hint at a fatty heart, as, feeble and intermitting action of the heart, partly relieved by walking; heart's beats wanting in energy and its sounds distant and feeble; action of heart slow and fluttering; pulse small and slow. We know that Brom. and its preparations are more suitable to persons with blue eyes and light hair, and it is a well-known fact that blondes are more given to obesity than brunettes, and the fatty heart in young persons may thus find its simillimum in the Bromides.

We have often remarked that the salts of Magnesium are too often neglected, and only since Schüssler led our attention to their great value, we find them more frequently reported. We have often heard of cases where the salts of Lime failed to eradicate that unknown psora, while the salts of Magnesium acted splendidly. We find in both slow dentition, with bloatedness in abdomen, but in the former more diarrhœa, whereas the latter has constipation. *Magnesia muriatica* has oppressed breathing, greater after a meal, constrictive pain in chest and scapulæ; stitches in the heart, arresting the breathing; palpitation of the heart, when sitting, better from motion; violent palpitation, with pulsation in all the arteries; tension across the chest, coming from the pit of the right axilla. Guernsey gives as characteristic indications: sleeplessness, knotty, hard, difficult, and insufficient stools, crumbling to pieces as they pass the anus, and frequent fainting attacks, which seem to start from the stomach. Its uterine symptoms prove that it is worth our consideration in the fatty heart of women with the three F's—fair, fat, and forty. Where the second stage merges into the degenerative one, Magnesia phosphorica of Schüssler might do something, and it is a pity that we have no proving of it.

Will *Ferrum phosphoricum*, the Aconite and Belladonna combined of Schüssler, do something in such cases of obesity? Arndt and Kane mention Ferrum, and we know there is such a thing as a plethoric chlorosis as well as an anæmic chlorosis, with great palpitation of the heart and dyspnœa and cardialgia. Such chlorotic women sometimes show a false plethora, may even develop a pretty good panniculus adiposus, and, in spite of all their wailing and ailing, they get no commiseration, for they look so well. In correspondence with other symptoms, we ought to think here of the Phosphate of Iron.

Kapka recommends Arnica; Arndt, Lycopodium and Grapite; Kane, Arsenicum, Calcarea carb., Aurum mur., Digitalis, Ferrum, Phosphorus, and Sulphur, and we acknowledge our blindness that for many of these drugs we fail to see their applicability to the fatty heart.

At any rate, poor people need not go to Marienbad. Hygienic rules can be everywhere observed, and our materia medica does the rest. S. L.

